# A Singular Case of Purpura and Tetanus

**Published:** 1869-04

**Authors:** Edward R. Kittoe


ARTICLE XVI.

### A SINGULAR CASE OF PURPURA AND TETANUS.

Extract from an Inaugural Thesis presented to the Faculty of Chicago Medical College for Session of 1868–69. BY EDWARD R. KITTOE.

As you will see by the heading, I intend to proffer as my thesis a report of a singular case of purpura and tetanus, which I had the good fortune to see and watch from the first attack to the final recovery, during the first year of my study with my preceptor, which happened to be my father, and I made it a part of my study to visit with him some of the most important cases which came under his care. The one I now intend to give a report of, struck me at the time as being one of peculiar interest, and I wanted him to keep a record of it and make a report to some of the medical journals, but he neglected to do so, and I nôw take it up to lay before you, as I remember it, with the few notes I have been able to collect from the patient's father, as well as those of my preceptor. It was as follows: Philip Bolinger, aged 9 years, had what appeared to be a small pimple on the lower eyelid of the left eye, his father punctured it, and there escaped a small drop of pus; a few hours after it commenced to bleed very freely. Having tried in vain to arrest the hemorrhage, by all the means they had at hand, my preceptor (Dr. E. D. Kittoe) was called in. He found a clot of blood about the size of an ounce ball, which, on being touched, blood appeared all over it in drops like dew. He removed the clot and applied some Monsel's styptic on cotton, but with no effect. He applied successively, tannin, alum, kino, agaric, nitrate of silver, etc., etc., with no benefit. It being impossible to apply pressure with any direct force upon the wound, a bandage was passed around the head, with a compress, over the temporal artery. This appeared to answer the desired purpose for about twenty-four hours, at the end of which time, there being much tumefaction of the face and scalp of the affected side, with an erysipelatous blush, it was deemed unsafe to continue pressure any longer. Upon removing the bandage and dressings, blood immediately began to ooze out around the coagulum, which had gradually increased until now it was the size of a hen's egg, flattened out by the pressure. Upraising carefully the edge, blood streamed out rapidly, and he found that there was a slough involving nearly two-thirds of the eyelid. He then removed the entire clot, and endeavored to staunch the blood by styptics once more, but with very poor success. He then tried the application of ice water, by the way of irrigation, by means of a fruit can, with a minute hole in the bottom, suspended so as constantly to drop on the part affected. In the meantime the boy was put upon tonic treatment. There had appeared on various parts of his body, petechia. He was directed to take quinia sulph. gr. ss.; tr. ferri chlo. min. xx.; vini rubri ℥ss., every three hours, with beef essence, chicken soup, etc., *ad libitum.* After about twelve hours the ice-water treatment failing to arrest the bleeding, and the slough appearing to increase, recourse was had to Peruvian bark, and powdered charcoal mixed quite stiff with yeast, and applied to the wound. On the removal of the fourth poultice the entire slough came away, leaving a clean granulating surface, with a very minute arterial jet near the centre. This was grasped with a pair of Liston's artery forceps, and pretty effectually twisted, after which no further hemorrhage occurred, and the boy recovered rapidly. In about six weeks from the first attack of hemorrhage, he got a slight scratch on the temple on the same side, the bleeding from which was almost as unmanageable as at the first, but was controlled mainly by large doses of tinct. ferri chloridi, 25 drops every four hours, with port wine. Styptics, and pressure were again tried, but but with no avail. The bleeding was evidently controlled by the tinct. ferri. There was at the time profuse hemorrhage from the gums, but no appearance of petechia on any part of the body. The iron and wine together, with nutritious food, was continued for several weeks, when the boy appeared to recover perfectly his color and health.

About the 9th of December following this, (the first attack being in July,) he fell down stairs and cut his hand across the ball of the thumb, with a piece of glass. It was tied up, and gave no inconvenience until the third or fourth day, when it became painful and commenced bleeding very profusely. My preceptor was at once called on, and again tried the use of styptics, but with no success. He also placed a compress of cork over the radial artery, which did partially control the bleeding, but immediately a large slough began to form about the cut, and a very considerable coagulum, about the size of a hen's egg, formed over the first incision. This clot had very much the appearance of fungus hæmatodes, the blood oozing from it at innumerable points. He removed this once or twice and applied dry lint, which would appear to keep it in check for a time, for several hours. However, this was soon abandoned for the bark, charcoal and yeast, with the tinct. ferri, 25 drops every three hours, which again had the effect to check the sloughing, and eventually the hemorrhage. (I should also mention that he tried at this time the use of bromine and also carbolic acid, but without benefit.) Now came on another phase of the case. My preceptor was called in the night to see his patient, the father stating that the boy was in a "fit." On arriving at the house, he found that he had a regular attack of tetanus. The jaws were tightly clenched, and the spasms, which came on in paroxysms, were terribly severe. Perspiration poured from every pore, and the whole appearance of the child was distressing in the extreme. He at once gave him the sixth of a grain of morphine, and directed the same quantity to be given at intervals of an hour, until relief was afforded; to continue the wine, with a teaspoonful of tinct. cinchona comp., every three hours. After a lapse of twenty-four hours, no benefit appearing to arise from these means, and the spasms being perfectly terrific, the dose of morphine was increased to one-fourth of a grain, in combination with valerianate of ammonia, ʒi, repeated every hour; porter or lager beer *ad libitum.* The spasms continued with great severity for sixteen days, and the dose of morphine was eventually increased to half a grain every two hours. The bowels were kept open by enemas of mutton or chicken broth, administered every second day. At one time he tried bromide of ammonia, with no effect; also the bromide of potassa, with similar results. The spasms, after the sixteenth day, began gradually to subside, and after a lapse of twenty-six days left him entirely. The hand healed kindly during the first three or four days of the attack of tetanus, and the boy recovered rapidly.

There appeared to be a peculiar hemorrhagic tendency about this case. He is of a leucophlegmatic temperment, rather large, and somewhat fatter than usual for boys of his age. His mother died of phthisis pulmonalis. He had had an attack of scarlatina auginosa, about three months previous to the first attack of hemorrhage, but there was none of the sequelæ common in such cases, although he was much enfeebled by the disease, and continued peevish and fretful. The scarlatina, I am inclined to believe, acted as a predisposing cause to the purpura. Wood mentions it as one of the causes that sometimes produce the disease, and in this case it seems very plausible to consider it so, as previous to the attack of scarlatina there was none of the tendency to hemorrhage, but immediately after, the father noticed a tendency to ecchymosis upon the child's receiving the least bruise, so that I am led to believe that the purpura really followed immediately after, but was not discovered until the hemorrhage took place.

The pimple, as the father supposed it to be, I believe was one of the petechial spots in the form of a bloody blister, and that he was mistaken in regard to the escape of pus from it when he punctured it, as he was not aware that there was any petechia upon the body of the child until my preceptor showed them to him. There was but one crop of them, for as soon as the hemorrhage appeared they ceased to show themselves. At the time of the second attack, the child appeared to be in good health, with the one exception that there still remained a slight tendency to eccymosis, but upon his receiving the scratch upon the temple, the tendency to hemorrhage of the same severe form again showed itself. Of course, as would be naturally supposed, after the child recovered from two severe hemorrhages of fourteen days each, he was very much debilitated, and almost bloodless, for I have seen him loose almost a pint of blood at one dressing of the wound, the hemorrhage was so severe. He was, I think, the whitest piece of humanity I have ever seen. The third attack, which was only about two months after the second, hardly time for him to regain his strength, was, as I have said, caused by a slight cut from a piece of glass. The same hemorrhagic tendency still remained, and was equally as unmanagable, and, like its predecessors, lasted just fourteen days. Then came the attack of tetanus as soon as the hemorrhage was stopped and the hand began to heal. The cause of which was, without a doubt, the debility arising from the hemorrhage. In the paroxysms he ground out two of the back teeth of the left side and bit his tongue fearfully, the blood from which formed in a pretty firm clot, so that it had to be removed by a pair of forceps, for fear of its suffocating the child, and I am sure that any one would have thought just as all who saw him, that he could not possibly live through these spasms, now that he was so reduced, and yet they continued in their most severe form for sixteen, and in all twenty-six days, when, as I have said, the child recovered, and is now as healthy a looking boy as one might wish to see.

There is one or two things in the treatment of this case worth notice. First. I have neglected to say that the wounded hand and arm were put in a bowl of warm water as soon as a spasm came on, and it afforded great relief. Second. The cessation of the hemorrhage, upon the application of the bark, charcoal and yeast poultice, after having resisted all other known means; and Third. The benefit derived from the very large doses of tinct. ferri chloridi, which seemed to be the only means of checking the hemorrhage. He took as much as 25 drops every three hours; besides this the enormous quantities of wine and whiskey, as well as quinia and morphine, all having the desired effect.

The recovery of the patient seems to me almost a miracle, and is at least very remarkable. It is one of those cases that go to show us how much our patients will go through and still recover, and that while life still remains, we may yet hope to afford relief, or even better, see our patient recover his health entirely, a pleasure which to the medical man is so great that none but those who have experienced it can realize the joy it affords him. He feels, in such a case, that he has gained the great object that prompted him to study medicine, that of relieving his fellow-creature from suffering, or, as it were, rescuing him from the grave. A man who gains such triumphs as this over the greedy hand of death, has in my mind the most noble calling God has given to any of his creatures.

